# Lifestyle Behavior Interventions for Preventing Cancer in Adults with Inherited Cancer Syndromes: Systematic Review

**DOI:** 10.3390/ijerph192114098

**Published:** 2022-10-28

**Authors:** Celia Diez de los Rios de la Serna, Paz Fernández-Ortega, Teresa Lluch-Canut

**Affiliations:** 1 School of Nursing, Faculty of Medicine and Health Sciences, Bellvitge Campus, University of Barcelona (UB), 08907 Barcelona, Spain; 2 Institut Català d’Oncologia (ICO) Barcelona, Bellvitge, 08908 Barcelona, Spain

**Keywords:** cancer prevention, lifestyle intervention, hereditary cancer, risk reduction, high-risk cancer, health behaviors

## Abstract

(1) Background: The link between lifestyle behaviors and cancer risk is well established, which is important for people with personal/family history or genetic susceptibility. Genetic testing is not sufficient motivation to prompt healthier lifestyle behaviors. This systematic review aims to describe and assess interventions for promoting healthy behaviors in people at high risk of cancer. (2) Methods: The review was performed according to PRISMA guidelines using search terms related to hereditary cancer and health education to identify studies indexed in: CINAHL, MEDLINE, PubMed, Cochrane Library, Scopus, and Joanna Briggs, and published from January 2010 to July 2022. (3) Results: The search yielded 1558 initial records; four randomized controlled trials were eligible. Three included patients with and without a personal history of cancer who were at increased risk of cancer due to inherited cancer syndromes, and one included people undergoing genetic testing due to family history. Interventions targeted diet, physical activity, and alcohol. (4) Conclusions: There is a paucity of research on interventions for promoting healthy lifestyle behaviors in people with a high risk of cancer. Interventions produced positive short-term results, but there was no evidence that behavioral modifications were sustained over time. All healthcare professionals can actively promote healthy behaviors that may prevent cancer.

## 1. Introduction

Cancer is a multifactorial disease resulting from a combination of genetic and external factors [1], and it is projected to eventually become the leading cause of death in every country in the world [2].

**Figure 1:**
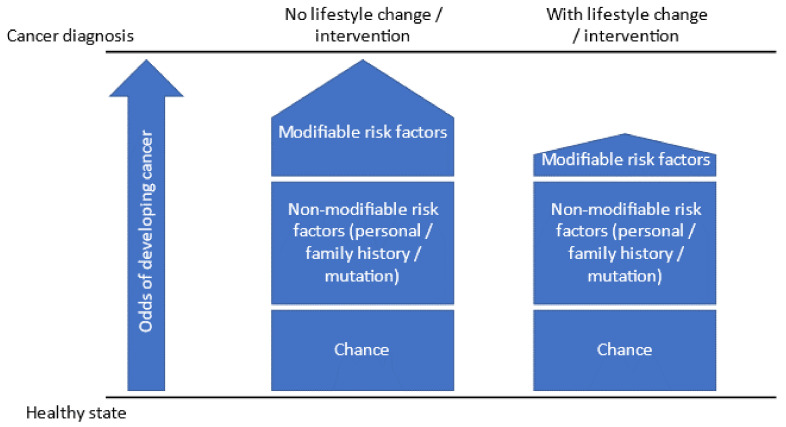
Addressing behavioral factors in people with increased risk for cancer can decrease their odds of developing cancer.

Cancer has a clear relationship with modifiable risk factors such as obesity, alcohol, and tobacco [3], and with partially modifiable factors such as environmental exposures and hormones [4]. Around 5–10% of the population has a very high risk of cancer due to inherited mutations [5], and in this group, the relationship between modifiable risk factors and cancer is more pronounced than in the general population [6,7,8] (Figure 1). For example, obesity can increase the risk of colorectal cancer by 49% in people with a genetic mutation [7]. Likewise, a systematic review found that drinking alcohol and being overweight increased breast cancer risk in BRCA carriers, while physical activity reduced it [9]. A prospective cohort study estimated that physical activity can reduce breast cancer risk in women with BRCA1 and BRCA2 by approximately 20% [8]. Therefore, modifying non-genetic risk factors related to behaviors or hormones can help decrease the relative risk of cancer [10].

**Figure 2:**
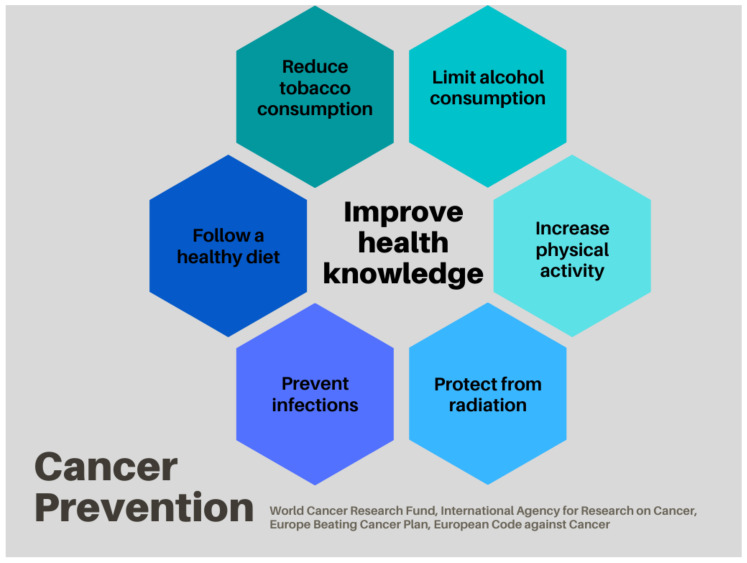
All cancer prevention strategies described can be grouped into these strategies. The figure is based on recommendations from the World Cancer Research Fund, International Agency for Research on Cancer, Europe Beating Cancer Plan, and the European Code Against Cancer [13,14,15,16].

Cancer prevention interventions constitute the best approach for reducing incident cases and known risk factors, and in turn the morbidity and mortality of some diagnoses [11,12] (Figure 2). However, these campaigns tend to be population-based rather than targeted to risk groups. Some patients’ associations and organizations for people affected by inherited cancer syndromes such as Facing Hereditary Cancer Empowered (www.facingourrisk.org, accessed on 9 September 2022) or AFALynch (afalynch.org, accessed on 9 September 2022) do organize campaigns and programs to improve health literacy, with the main aim of enabling people to make healthier lifestyle decisions and empower them to manage their personal cancer risk.

### 1.1. Genetic Counseling

Those who carry an increased risk of cancer due to their personal or family history are normally referred for genetic counseling for predictive testing. According to the Transnational Alliance of Genetic Counseling, the main aim of these consultations is to help patients understand their individual risk and make a decision about whether genetic testing is appropriate for them [17]. Counselors may also assess patients’ lifestyle and educate them on how to adapt to their cancer risk by reducing behavioral components [18], although there is no consensus on their precise role in providing advice about lifestyle behaviors [18].

Indeed, when a patient has an inherited cancer syndrome, genetic counseling focuses more on cancer screening and preventive surgery than on health education [19,20,21]. Thus, following these consultations, patients are more likely to increase their cancer surveillance or opt for risk-reducing surgeries than to change behaviors [22]. The studies do not explain whether this is due to lack of awareness and information provided during counseling or because these interventions reduce their perception of risk [21]. A systematic review [22] of lifestyle behaviors in patients receiving genetic counselling found that communicating the risk of cancer due to genetic alteration has little impact on lifestyle behaviors such as smoking, diet, or physical activity. However, a review evaluating interventions during colorectal and breast cancer screening found that behavioral interventions can promote increased physical activity and dietary modifications [23]. The same tendency has been observed in cancer survivors, who are motivated to engage in interventions following treatment; however, these changes are not normally sustained long term [24,25].

### 1.2. Changing Lifestyle Behaviors

Using all the information of an individual’s known risk factors, including their behavioral habits, is necessary for a personalized approach. Assessing individuals’ risks, motivations, and priorities gives people the opportunity to self-manage their risk [6]. However, knowledge alone is insufficient for effective cancer prevention; it must be supplemented with health education interventions that favor behavior change [26].

People with an increased risk of cancer seek advice from different healthcare providers, but these professionals may miss opportunities to provide information and motivate individuals to change health-related behavior [27]. Family physicians, nurses, and other health professionals often lack proper risk assessment and communication skills [21]. The precise impact of health interventions on health behavior in patients at high risk of cancer due to inherited cancer syndromes remains unquantified.

Improving awareness on this important topic would support the identification and planning of interventions tailored to these individuals’ needs and empower them to reduce risky behaviors, thereby improving overall cancer morbidity, survival, and the patient experience.

This systematic review was conceived to address this gap in knowledge by identifying and evaluating interventions for promoting healthy lifestyles in people with a high risk of cancer due to inherited cancer syndromes.

The primary aim is to assess the effect of health education interventions for modifying lifestyle behaviors in adults with a high risk of cancer. The secondary aim is to identify the healthcare professionals responsible for the interventions and describe motivations and barriers for change.

## 2. Materials and Methods

This systematic review followed the Joanna Briggs Institute methodological guidelines [28] and was reported according to the Preferred Reporting Items for Systematic Reviews and Meta-Analyses (PRISMA) statement guidelines [29]. The review was registered in 2020 on PROSPERO: CRD42020209921 (PRISMA checklist included in Supplementary Table S1).

The research question was formulated using the PICO typology [30]: P—Population: adults at increased risk of cancer; I—Intervention: health education interventions; C—Comparison: no intervention; O—Outcome: modification of lifestyle behaviors.

Patient and public involvement: Input from public and patient involvement informed this research. Specifically, three people with genetic syndromes (one with BRCA unaffected by cancer, and two with Lynch syndrome—one with cancer and the other without) were involved in the project development phase and provided feedback on the appropriateness and pertinence of the objectives to the population under study.

### 2.1. Search Strategy

A systematic search was conducted in the following electronic databases: Ebsco CINAHL, Ovid MEDLINE, PubMed, Cochrane Library, Scopus, and Joanna Briggs. Relevant peer-reviewed studies published from January 2010 to August 2022 were included, as genetic counseling related to cancer risk only began in the late 1990s, and published studies on behavioral risks in these populations did not begin to appear until the 2010s [18]. References cited in systematic reviews evaluating lifestyle interventions [23,31,32] in other populations were screened for additional articles which might have been overlooked. The website clinicaltrials.gov was also checked for any published protocols or feasibility studies.

The search strategy combined the key PICO terms using free text and MeSH terms related to cancer, hereditary cancer, and health education and promotion (Supplementary Table S2). A university librarian was consulted to validate the search strategies.

The searches were limited to research articles published in English, Portuguese, or Spanish (see Supplementary Table S3 for an example of a database search).

### 2.2. Eligibility Criteria

All intervention studies that met the inclusion criteria and were published in peer-reviewed journals were evaluated. 

Inclusion criteria were based on the research question and study objectives:

- Studies focused on adults with a high risk of cancer, defined as those with a significant personal or/and family history of cancer undergoing genetic testing or confirmed inherited cancer syndrome [5].
- Studies evaluating the effects of behavioral interventions.
- RCTs and other experimental studies researching the effect of health education interventions in this population (randomized trials and non-randomized trials) with or without a control group (experimental studies comparing the intervention vs another form of intervention as comparator), and written in English, Portuguese, or Spanish.
- Articles were excluded if they were:
- Studies of unmodifiable factors such as genes.
- Studies not assessing behavioral interventions (for example evaluating the effect of medication or screening).
- Studies in people receiving active treatment for cancer, as they experience different cancer-related barriers and have different motivation towards interventions that improve quality of life or symptoms rather than reducing risk [33].
- Expert and medical society recommendations, editorials, reviews, and commentaries.
- Study protocols, case reports, or drug trials.
- Studies performed only in animals.
- Studies that exclude patients with genetic mutations.

### 2.3. Screening

The principal investigator (PI) performed an initial screening of titles for all records retrieved by the search. Potentially relevant publications were downloaded into reference management software and de-duplicated. The PI screened the abstracts against the eligibility criteria, and then two authors independently read the full text of the remaining articles to determine whether they met the review’s inclusion criteria.

### 2.4. Assessment of Methodological Quality and Bias

The Cochrane RoB2 tool was used to assess the risk of bias in the included RCTs [34]. This tool is used to rate each specified outcome as being at low risk, causing some concerns, or having a high risk of bias. Quality was assessed using the Joanna Briggs Institute critical appraisal tools for RCTs [35]. No studies were excluded based on these assessments.

### 2.5. Data Extraction and Synthesis

The PI extracted data into a customized evidence table in Excel, and the second author double-checked them. The data extraction form was piloted using the first studies to define what information to collect and ensure comprehensive data capture. Data included study characteristics, population, lifestyles addressed, description of the intervention, and the measures of efficacy, based on the Template for Intervention Description and Replication (TIDieR) checklist for reporting interventions [36]. In addition, we noted which professionals delivered the intervention along with motivations and barriers for behavioral modification and engagement.

The results were combined in an organized, visual table, where comparable results can be pooled as recommended by the Centre for Reviews and Dissemination (CRD) guidance for undertaking reviews in healthcare [30].

Intervention studies involving animals or humans, and other studies that require ethical approval, must list the authority that provided approval and the corresponding ethical approval code.

## 3. Results

**Figure 3:**
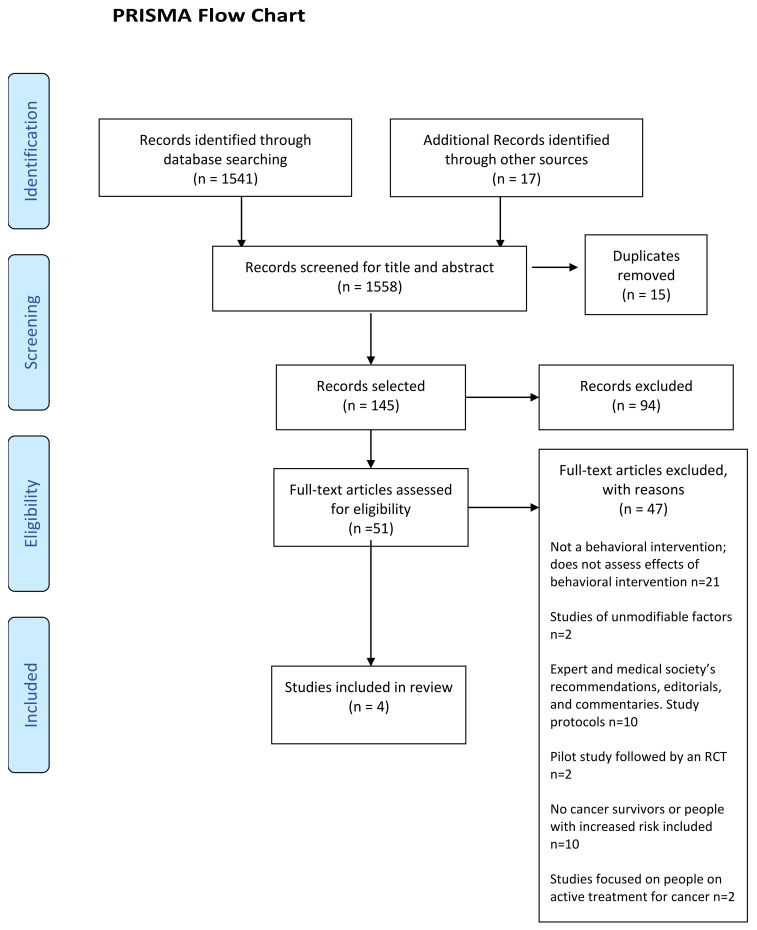
PRISMA diagram of the article selection/screening process (adapted from [28]).

The initial database search yielded 1558 records. After screening titles and abstracts, 51 publications were retrieved for full-text review, and four RCTs met our selection criteria (PRISMA flow charts; Figure 3).

### 3.1. Characteristics of Included Studies

Table 1 summarizes the main characteristics of included studies, which all took place in Europe (one each in the UK, Germany, The Netherlands, and Italy). All were written in English. 

The samples sizes ranged from 29 to 502 adult participants, with a mean age of 41 [37] to 49 years old [38] (range 24 to 72). One study’s (25%) primary outcome was changes in lifestyle behavior. Kiechle et al. [37] and Bruno et al. [39] studied patients with the BRCA mutation, Vrieling et al. [40] studied patients with Lynch syndrome, and Anderson et al. [38] included people with a family history of breast or colorectal cancer prior to genetic testing. One study included only healthy individuals with a high risk of BRCA or Lynch [38], one only BRCA carriers with a personal history of cancer [37], and the other two populations with hereditary alterations (Lynch syndrome [40] and BRCA carriers [39]), with or without a personal history of cancer.

### 3.2. Overall Methodological Quality of the Studies and Risk of Bias of the RCTs 

The methodological quality of the studies was particularly affected by the lack of blinding in participants and in the professionals delivering the intervention. It was also unclear whether the assessors were blinded to the trial arm (Table 2).

**Figure 4:**
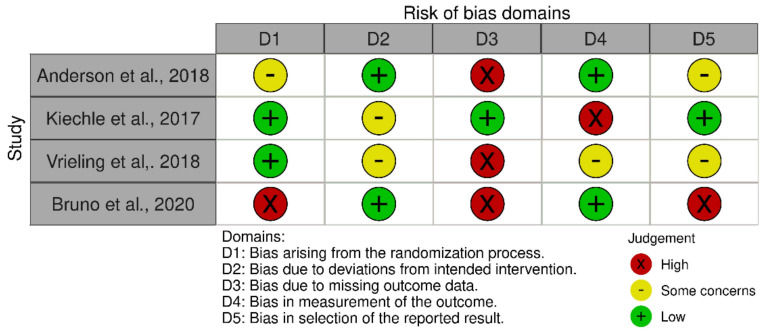
Risk of bias of included studies [14] Studies: Anderson et al. [38], Kiechle et al. [37], Vrieling et al. [40] and Bruno et al. [39].

The risk of bias assessment showed that all RCTs either caused some concerns or were at high risk of bias (Figure 4). 

Randomization and allocation concealment were reasonably well described for most studies, as were outcomes and reasons for participant attrition. Logically, most participants were aware of the intervention they were allocated to, but most studies did not clarify if the outcomes assessors were blinded to treatment assignment.

### 3.3. Interventions

All four included studies that targeted diet, while three also assessed interventions to increase physical activity. Alcohol intake was targeted in two studies but was not reported in either. One study mentioned tobacco but did not report results.

Regarding the type of intervention, one study used a combination of dietary activities and cooking classes [39]; one, information delivered by leaflets [40]; and two, information provided through consultations and education [37,38]. 

Interventions were somewhat different in terms of the mode of delivery, duration, and the nature of the intervention. All four studies had some kind of face-to-face sessions, and in two these were complemented by remote contacts via email or telephone. Interventions lasted from 1 to 6 months. All studies assessed outcome variables at baseline and post-intervention, and two also included a follow-up measurement to determine whether the changes were maintained at 5 months [40] and 9 months after the intervention [37]. 

Anderson et al. [38] described assessing adherence to the protocol by recording and analyzing a random sample of visits and telephone calls. This study also included information about the behavior change models and theories on which their intervention was based, specifying the behavioral techniques, such as goal setting, used in the interventions [38] (Table 3). 

Studies were led and monitored by healthcare professionals from diverse backgrounds, including nurses, genetic counsellors, and others. All recruited participants who were attending a genetic counseling unit, but only one study indicated that genetic counsellors led the intervention [40]. One study specifically mentioned that the intervention was nurse-led [38]; the rest did not specify which professionals delivered it [37,39]. 

### 3.4. Outcomes

Table 4 details the results of included studies according to the behaviors targeted.

#### 3.4.1. Diet

Dietary behavior was measured using different questionnaires; two studies [37,39] used the Mediterranean Diet Adherence Screener (MEDAS) [41], and three used different self-reported questionnaires about adherence to recommendations [40] or sections of these [38,39]. Despite differences in data collection methods, the results had commonalities across the studies, with reports of increased fruit and vegetable intake, and, where measured, an increase in fiber and a reduction in red meat intake. Dietary behaviors improved at the post-intervention time point, but the magnitude of the effect showed a sensible decline on follow-up measurements, although they remained better than baseline levels [37,38,40].

#### 3.4.2. Physical Activity

Three studies assessed self-reported physical activity, with two reporting that participants performed more minutes of moderate physical activity at the post-intervention time point [38]. Another study objectively measured physical activity with monitors such as pedometers [38]. Kiechle et al. [37] chose to measure aerobic capacity (VO_2_) as an objective measure of resistance to physical activity.

All studies showed an increase in physical activity, using different outcomes. The post-intervention assessment showed that people increased their physical activity; however, participants who were assessed over follow-up tended to regress towards baseline levels [37].

#### 3.4.3. Weight/Body Mass Index (BMI)

Results on weight and BMI differed between studies; two studies did not report differences between the intervention and control group [37,40], while two studies reported more weight loss and lower BMI in the intervention group [38,39].

#### 3.4.4. Alcohol and Tobacco

Two studies mentioned alcohol [38,40] but did not report outcomes from the intervention. One study also included tobacco among the targets of the intervention [38], but there was no mention of measures or changes.

#### 3.4.5. Motivations and Barriers

The included studies did not assess factors such as motivation for change, readiness for change, or patients’ mental health. The feasibility studies showed good motivation and satisfaction with the intervention but reported barriers to adherence, such as duration, travel needs, and personal barriers [37,38]. Some participants also dropped out due to family commitments [39] or lack of motivation [37].

## 4. Discussion

The findings of this systematic review suggest that healthcare interventions can be useful to modify lifestyle behaviors in adults with a high risk of cancer. However, current evidence is scarce and highly skewed towards interventions for people with a personal history of cancer. Behavioral modifications were not the primary objective of the studies, half of which assessed other parameters (blood test results, awareness of recommendations, acceptability of the intervention) as primary outcomes.

While there is extensive evidence supporting the relationship between modifiable lifestyle behaviors and cancer [3,42], included studies assessed only a few behavioral factors, mainly diet and physical activity. Alcohol intake is associated with the risk of breast, colorectal, liver, and other cancer [43], but it was not addressed in the included articles. This was also the case for tobacco use.

The included studies showed that lifestyle interventions are effective in driving people to increase their physical activity and improve their diet, but these improvements are not sustained over time, regressing towards baseline or remaining slightly better in the case of dietary modification. The findings were not conclusive for weight/BMI changes, as some studies showed no changes and others a greater weight loss in the intervention group.

This review reveals evidence gaps around behavioral interventions in people with a high risk of cancer, especially in those without a personal history of the disease. All studies included some cancer survivors, except Anderson et al., 2018 [38], who included only healthy participants with a family history of cancer. Evidence suggests that people with a confirmed or suspected high risk of cancer do not take the initiative in seeking behavioral recommendations from healthcare professionals [39]. Patients are often unaware or have incorrect perceptions about behavior and cancer risk [19]. In a study that took place in a breast screening service, alcohol was identified as a risk factor by only 19.5% of healthy women attending the screening and by less than half of the healthcare professionals working there [12]. In the study by Vrieling et al. [40], knowledge of cancer risk factors was significantly greater in the intervention versus the control group. Risk perception influences behavior [44], so the lack of awareness of the importance of behavioral factors among both patients and healthcare professionals is concerning, undermining effective behavior change.

The studies included in this systematic review involved participants who were already motivated (i.e., they agreed to join studies focused on modification of lifestyle behaviors) but still identified some barriers to change. However, none of the included studies addressed these barriers or explored the participants’ motivations. Because behavior change is a complicated process [45], designing a behavioral intervention requires understanding the behavior and identifying the intervention options, including the individual’s motivation and capacity for change (a process well defined in the Behavior Change Wheel [46]), which the included studies failed to do. One study [38] mentions the behavior change models and the reasoning underpinning the intervention, but none mentioned previous studies supporting the intervention options in relation to behavior change.

The failure to use behavioral techniques or models may also explain the heterogeneity of behavioral interventions in the included studies used leaflets, activities, and consultations. Systematic reviews and studies have compared face-to-face, online, and blended interventions, without a clear preference [47,48,49]. While the included studies showed some evidence of effecting behavioral changes or even improving awareness and knowledge of cancer risk factors (such as the study by Vrieling et al. [40]), they failed to plan the interventions using models to sustain these changes. The use of behavior change methods and theories to design interventions, together with public and patient involvement during the design and implementation phases, are of critical importance in healthcare interventions. In the context of hereditary cancer, such approaches have proven useful in improving health-seeking behaviors associated with the detection of Lynch syndrome [50].

Patients with a high risk of cancer live with the extra psychological burden of uncertainty about when and if they will develop a cancer [51]. They may be motivated to change, but the goal of reducing cancer risk is not sufficient to maintain these changes over time. Promoting self-care and good mental health favors sustainability [52]. Healthcare professionals should consider these complex needs and build their skills in behavioral interventions to support this population.

Whether behavioral interventions in people at high risk of cancer have a true impact on cancer incidence remains unknown. Just two studies assessed outcomes at follow-up, making it difficult to assess effectiveness over time.

Although genetic counsellors do meet with people at high risk of cancer, they have little opportunity to discuss behavioral factors, as they normally see their patients only twice [18]. Ideally, all health professionals (i.e., genetic counsellors, oncologists, nurses) should be involved in care for people with a high risk of cancer, initiating conversations about lifestyle behaviors and offering evidence-based recommendations on behavioral modifications, also considering the psychosocial support needed to achieve them. This review found different healthcare professionals involved in delivering the interventions, suggesting that there may not be any specific healthcare professionals with the responsibility for addressing lifestyle behaviors in people with inherited cancer syndromes and raising the need to define competencies in that regard [53,54].

### Strengths and Limitations

This review included four RCTs of medium or low quality. Due to the limited number of studies in this field, no studies were excluded due to quality, but the quality of the included studies constitutes a limitation.

A further limitation is the heterogeneity of methods and interventions; each study measured different behavioral outcomes in different ways, precluding meta-analysis. Results were instead combined in a narrative synthesis and a table.

The main weakness of the studies was insufficient blinding in participants and unclear blinding in outcome assessors. However, blinding patients in this kind of interventions is not possible, as they have to actively participate in the intervention, so they know which group they belong to. This could also influence their answers on the self-assessment questionnaires. However, this limitation is not inherent to researchers performing the pre- and post-intervention assessments, who could more easily be blinded. 

Including people both with and without a history of cancer poses a limitation. Interventions may have different effects in people with a family history of cancer or an inherited cancer syndrome compared to people with a personal history of cancer, who generally make more lifestyle behavior changes and whose motivation is more about improving quality of life than reducing risks [24]. The studies that included both people with and without a personal history of cancer did not separate or compare the results between populations, precluding any differentiated analysis in our study.

Another limitation is that the results are based on experimental contexts, making the conclusions of the studies more difficult to extrapolate to general practice, as all interventions had specific funding that may be difficult to sustain on finishing the study. 

The main strengths of this review were that the included studies were adequately randomized, had comparable groups, and used similar interventions. Added strengths include the review’s clear protocol, methods, and the inclusion of six different databases in the search. A variety of studies were included in the review, providing a broad overview of the types of behavioral interventions applied to reduce cancer risk. The review also identified gaps in knowledge and highlighted areas for future research.

## 5. Conclusions

There are few studies of behavioral interventions for people with a high risk of cancer, and most of these are focused on diet and exercise. The interventions explored showed that behavioral interventions promote positive short-term results, but they fail to promote long-term lifestyle modification.

Future research and interventions should focus on healthcare professionals’ knowledge of the impact of behavior on cancer risk, as well as behavior change techniques and promotion of mental health. Strengthening the competencies of healthcare professionals in this regard can help in identifying and supporting the needs of people at high risk of cancer.

## Figures and Tables

**Table 1:** Characteristics and methodology of the studies.

Study	Design, Country	Sample Size	Population	Intervention	Comparator	Duration	Outcomes		
							Primary	Secondary	Measurements
Anderson et al., 2018 [38]	2-arm RCT (feasibility), UK	N = 78 (intervention n = 39, control n = 39)	People with family history of breast or colorectal cancer and BMI of ≥25 kg/m^2^	Face-to-face session plus 4 telephone consultations, pedometer, and walking program	Usual care	3 months	Feasibility measures	Changes in weight, physical activity, diet, psychosocial measures	Changes in weight: kg, waist circumference and BMI. Physical activity: IPAQ-Short and physical activity monitors (with sedentary time, moderate and vigorous activity, and step counts) Diet: Dietary Instrument for Nutrition Education questionnaire Alcohol: 7-day alcohol record
Kiechle et al., 2017 [37]	2-arm RCT(feasibility), Germany	N = 68 (intervention n = 33, control n = 35)	*BRCA1* or *2* carriers with cancer	Structured face-to-face behavioral intervention for increased physical activity and nutrition education	Lecture on the positive effects of PA and healthy diet	12 months (3 intervention, 9 supervision)	Adherence to and acceptability of the intervention	Effects on physical activity, diet, BMI, QoL, and stress	BMI Diet: MEDAS Questionnaire and eating habits, nutrient and fat calorie intake (EPIC-FFQ) Physical activity: maximal oxygen intake (VO_2_ peak), ventilatory threshold (O_2_ at VT1), and physical activity (IPAQ)
Vrieling et al., 2018 [40]	2-arm RCT, Netherlands	N = 226 (intervention n = 114, control n = 112)	People with Lynch syndrome with and without cancer	WCRF health promotion materials and information about colorectal cancer symptoms and prevention	Usual care	6 months (1 intervention, 5 follow-up)	Awareness of cancer risk factors	Adherence to WCRF recommendations	BMIWCRF/AICR adherence, Diet: adapted version of FFQ validated questionnaire Physical activity: Short Questionnaire to Assess Health Enhancing Physical Activity (SQUASH)
Bruno et al., 2020 [39]	2-arm RCT, Italy	N = 502 (intervention n = 254, control n = 248)	BRCA carriers, with or without a previous cancer	Dietary activities, cooking courses followed by lunch and nutritional conferences	Recommendations on cancer prevention	6-month intervention	IGF-I reduction	Food intake	Height and body weight Diet: MEDAS Questionnaire

AICR: American Institute for Cancer Research, BMI: body mass index, FFQ: food frequency questionnaire, IGF-I: insulin-like growth factor-I, IPAQ: International Physical Activity Questionnaire, MEDAS: Mediterranean Diet Adherence Score, RCT: randomized controlled trial, QoL: quality of life, WCRF: World Cancer Research Fund, VT1: ventilatory threshold 1.

**Table 2:** Quality assessment using the Joanna Briggs Institute critical appraisal tools for RCTs [35].

Questions for Critically Appraising the Quality of RCTs	Anderson et al. [38]	Kiechle et al. [37]	Vrieling et al. [40]	Bruno et al. [39]
Was true randomization used for assignment of participants to treatment groups?	Unclear	Yes	Unclear	Unclear
2. Was allocation to treatment groups concealed?	Yes	Yes	Unclear	Unclear
3. Were treatment groups similar at baseline?	Yes	Yes	Unclear	Yes
4. Were participants blind to treatment assignment?	No	No	No	No
5. Were those delivering treatment blind to treatment assignment?	No	No	No	No
6. Were outcomes assessors blind to treatment assignment?	Yes	Unclear	Unclear	Unclear
7. Were treatment groups treated identically other than the intervention of interest?	Yes	No	Yes	Yes
8. Was follow-up complete and if not, were differences between groups in terms of their follow-up adequately described and analyzed?	Yes	Yes	Yes	Yes
9. Were participants analyzed in the groups to which they were randomized?	Yes	Yes	Yes	Yes
10. Were outcomes measured in the same way for treatment groups?	Yes	No	Yes	Yes
11. Were outcomes measured in a reliable way?	Yes	Yes	Yes	Yes
12. Was appropriate statistical analysis used?	Unclear	Yes	Unclear	Yes
13. Was the trial design appropriate, and any deviations from the standard RCT design (individual randomization, parallel groups) accounted for in the conduct and analysis of the trial?	Unclear	Yes	No	Unclear

**Table 3:** Behavioral techniques and models used in each study.

Study, Country	Behavioral Techniques or Strategies	Behavior Change Models	Measures of Motivation or Barriers
Anderson et al., 2018 [38], UK	Identify what goals mean to participants Realistic goal-setting Implementation intentions	1. Leventhal’s self-regulatory theory 2. Social cognitive theory 3. Health action process approach	Beliefs about cancer cause and risk reduction Barriers and motivations
Kiechle et al., 2017 [37], Germany	Not stated	Not stated Mentions theory of planned behavior in a previous publication with the protocol but does not state if the intervention is based on it	N/A
Vrieling et al., 2018 [40], The Netherlands	N/A	Not stated Mentions behavior change theories and models in the Discussion	N/A
Bruno et al., 2020 [39], Italy	Not stated	Not stated in the study or previously published protocol	N/A

**Table 4:** Summary of findings across targeted behaviors or risk factors.

Study	Physical Activity	Dietary Intake	Weight/BMI	Alcohol/Tobacco	Other
Anderson et al. [38]	Increase in moderate exercise: 58.1 to 86.8 min in intervention group, 60.3 to 73.2 min in control; no significant difference in vigorous activity	Decrease in dietary fat scores (mean difference −7.8 in intervention group, −1.2 control) Change in fiber intake: +0.6 intervention group, −0.8 control	Mean weight loss: −3.2 kg intervention group, −0.3 kg control	Not reported	Barriers to change: daily routines, sedentary occupations, family commitments, poor physical or mental health, stressful events, complex relationships with food
Kiechle et al. [37]	VO_2_ peak improved in the intervention group at 3 months, but these effects diminished at 12 months. Aerobic capacity and min of exercise per week did not improve	No differences in the total daily calorie intake or fat intake in either group. Baseline median MEDAS score was 2 points higher in the intervention group versus control (*p* = 0.020); this difference widened significantly at 3 months (*p* = 0.001).	No significant differences between groups	N/A	Women with chronic stress were probably included. At 12 months, median scores on the Short Screening Scale for Chronic Stress were significantly lower in the intervention group compared to control (14.6 versus 20.9; *p* = 0.022). Health-related quality of life was similar between groups.
Vrieling et al. [40]	Adherence to physical activity recommendations improved in both groups	Adherence to the WCRF/AICR recommendations did not differ between groups. Highest adherence rates were found for intake of alcohol and sugary drinks.	No significant differences between groups	Not reported	Awareness and knowledge of the WCRF/AICR recommendations varied by recommendation but were significantly higher in the intervention group compared with the control group for all recommendations.
Bruno et al. [39]	N/A	Compared to control, the intervention group showed significantly increased intake of whole grain products (*p* < 0.001) and legumes, nuts, and seeds (*p* = 0.02), and reduced intake of dairy products (*p* = 0.01) and red and processed meat (*p* = 0.04)	More weight loss (*p* < 0.001) and lower BMI (*p* < 0.001) in intervention vs. control	N/A	Intervention group showed larger reduction in waist circumference (*p* = 0.01), hip circumference (*p* = 0.01), total cholesterol (*p* = 0.04), triglycerides (*p* = 0.01), and IGF-I levels (*p* = 0.02) compared to control.

N/A: not applicable, AICR: American Institute for Cancer Research, WCRF: World Cancer Research Fund, IGF-I: insulin-like growth factor-I.

## Data Availability

The review was registered on PROSPERO: CRD42020209921.

## Supplementary Materials

The following supporting information can be downloaded at: https://www.mdpi.com/article/10.3390/ijerph192114098/s1, Table S1: PRISMA Checklist of items to include when reporting a systematic review or meta-analysis [29]; Table S2: Free-text and MeSH terms pertinent to three strings of search. String A was first combined with string B, and subsequently with string C; Table S3: Example of PubMed search.

## References

[1] Wu S., Zhu W., Thompson P., Hannun Y.A. (2018). Evaluating intrinsic and non-intrinsic cancer risk factors. Nat. Commun..

[2] Ferlay J., Colombet M., Soerjomataram I., Mathers C., Parkin D.M., Piñeros M., Znaor A., Bray F. (2019). Estimating the global cancer incidence and mortality in 2018: GLOBOCAN sources and methods. Int. J. Cancer.

[3] Whiteman D.C., Wilson L.F. (2016). The fractions of cancer attributable to modifiable factors: A global review. Cancer Epidemiol..

[4] Marzo-Castillejo M., Vela-Vallespín C., Bellas-Beceiro B., Bartolomé-Moreno C., Melús-Palazón E., Vilarrubí-Estrella M., Nuin-Villanueva M. (2018). Recomendaciones de prevención del cáncer. Actualización PAPPS 2018. Aten. Primaria..

[5] Foulkes W.D. (2008). Inherited Susceptibility to Common Cancers. N. Engl. J. Med..

[6] Zhu M., Wang T., Huang Y., Zhao X., Ding Y., Zhu M., Ji M., Wang C., Dai J., Yin R. (2021). Genetic Risk for Overall Cancer and the Benefit of Adherence to a Healthy Lifestyle. Cancer Res..

[7] Lazzeroni M., Bellerba F., Calvello M., Macrae F., Win A., Jenkins M., Serrano D., Marabelli M., Cagnacci S., Tolva G. (2021). A Meta-Analysis of Obesity and Risk of Colorectal Cancer in Patients with Lynch Syndrome: The Impact of Sex and Genetics. Nutrients.

[8] Daniele A., Divella R., Pilato B., Tommasi S., Pasanisi P., Patruno M., Digennaro M., Minoia C., Dellino M., Pisconti S. (2021). Can harmful lifestyle, obesity and weight changes increase the risk of breast cancer in BRCA 1 and BRCA 2 mutation carriers? A Mini review. Hered. Cancer Clin. Pract..

[9] Coletta A.M., Peterson S.K., Gatus L.A., Krause K.J., Schembre S.M., Gilchrist S.C., Arun B., You Y.N., Rodriguez-Bigas M.A., Strong L.L. (2020). Diet, weight management, physical activity and Ovarian & Breast Cancer Risk in women with BRCA1/2 pathogenic Germline gene variants: Systematic review. Hered. Cancer Clin. Pract..

[10] Carr P.R., Weigl K., Edelmann D., Jansen L., Chang-Claude J., Brenner H., Hoffmeister M. (2020). Estimation of Absolute Risk of Colorectal Cancer Based on Healthy Lifestyle, Genetic Risk, and Colonoscopy Status in a Population-Based Study. Gastroenterology.

[11] Wild C.P., Espina C., Bauld L., Bonanni B., Brenner H., Brown K., Dillner J., Forman D., Kampman E., Nilbert M. (2019). Cancer Prevention Europe. Mol. Oncol..

[12] Sinclair J., McCann M., Sheldon E., Gordon I., Brierley-Jones L., Copson E. (2019). The acceptability of addressing alcohol consumption as a modifiable risk factor for breast cancer: A mixed method study within breast screening services and symptomatic breast clinics. BMJ Open..

[13] World Cancer Research Fund International (2016). Our Cancer Prevention Recommendations.

[14] Makarem N., Lin Y., Bandera E.V., Jacques P.F., Parekh N. (2015). Concordance with World Cancer Research Fund/American Institute for Cancer Research (WCRF/AICR) guidelines for cancer prevention and obesity-related cancer risk in the Framingham Offspring cohort (1991–2008). Cancer Causes Control.

[15] Schüz J., Espina C., Villain P., Herrero R., Leon M.E., Minozzi S., Romieu I., Segnan N., Wardle J., Wiseman M. (2015). European Code against Cancer 4th Edition: 12 ways to reduce your cancer risk. Cancer Epidemiol..

[16] McGuinness L.A., Higgins J.P.T. (2021). Risk-of-bias VISualization (robvis): An R package and Shiny web app for visualizing risk-of-bias assessments. Res. Synth. Methods.

[17] Abacan M.A., Alsubaie L., Barlow-Stewart K., Caanen B., Cordier C., Courtney E., Davoine E., Edwards J., Elackatt N.J., Gardiner K. (2019). The Global State of the Genetic Counseling Profession. Eur. J. Hum. Genet..

[18] Resta R.G. (2019). What have we been trying to do and have we been any good at it? A history of measuring the success of genetic counseling. Eur. J. Med. Genet..

[19] Quillin J.M. (2016). Lifestyle Risk Factors Among People Who Have Had Cancer Genetic Testing. J. Genet. Couns..

[20] Lammert J., Grill S., Kiechle M. (2018). Modifiable Lifestyle Factors: Opportunities for (Hereditary) Breast Cancer Prevention—A Narrative Review. Breast Care..

[21] Nippert I., Julian-Reynier C., Harris H., Evans G., Van Asperen C.J., Tibben A., Schmidtke J. (2014). Cancer risk communication, predictive testing and management in France, Germany, the Netherlands and the UK: General practitioners’ and breast surgeons’ current practice and preferred practice responsibilities. J. Community Genet..

[22] Hollands G.J., French D.P., Griffin S.J., Prevost A.T., Sutton S., King S., Marteau T.M. (2016). The impact of communicating genetic risks of disease on risk reducing health behaviour: Systematic review with meta-analysis. BMJ.

[23] Orange S.T., Hicks K.M., Saxton J.M. (2021). Effectiveness of diet and physical activity interventions amongst adults attending colorectal and breast cancer screening: A systematic review and meta-analysis. Cancer Causes Control.

[24] Grimmett C., Simon A., Lawson V., Wardle J. (2015). Diet and physical activity intervention in colorectal cancer survivors: A feasibility study. Eur. J. Oncol. Nurs..

[25] Spees C.K., Braun A.C., Hill E.B., Grainger E.M., Portner J., Young G.S., Kleinhenz M.D., Chitchumroonchokchai C., Clinton S.K. (2019). Impact of a Tailored Nutrition and Lifestyle Intervention for Overweight Cancer Survivors on Dietary Patterns, Physical Activity, Quality of Life, and Cardiometabolic Profiles. J. Oncol..

[26] Michie S., Atkins L., West R. (2016). The Behaviour Change Wheel. A Guide to Designing Interventions.

[27] Edwards A.G.K., Naik G., Ahmed H., Elwyn G.J., Pickles T., Hood K., Playle R. (2013). Personalised risk communication for informed decision making about taking screening tests. Cochrane Database Syst. Rev..

[28] Tufanaru C., Munn Z., Aromataris E., Campbell J., Hopp L. (2020). JBI Manual for Evidence Synthesis. JBI Manual for Evidence Synthesis.

[29] Moher D., Liberati A., Tetzlaff J., Altman D.G. (2009). Preferred Reporting Items for Systematic Reviews and Meta-Analyses: The PRISMA Statement.

[30] Centre for Reviews and Dissemination, Akers, J (2009). Systematic Reviews: CRD’s Guidance for Undertaking Reviews in Health Care.

[31] Underhill-Blazey M., Rodriguez D., Norton S.A. (2022). Scoping Review of Nonsurgical, Nonpharmacologic Interventions After Risk Reduction: Improving Quality of Life for Patients with Inherited Cancer Risk. Oncol. Nurs. Forum.

[32] Malakou E., Linardakis M., Armstrong M.E.G., Zannidi D., Foster C., Johnson L., Papadaki A. (2018). The Combined Effect of Promoting the Mediterranean Diet and Physical Activity on Metabolic Risk Factors in Adults: A Systematic Review and Meta-Analysis of Randomised Controlled Trials. Nutrients.

[33] André N., Pillaud M., Davoust A., Laurencelle L. (2018). Barriers Identification as Intervention to Engage Breast Cancer Survivors in Physical Activity. Psychosoc. Interv..

[34] Sterne J.A.C., Savović J., Page M.J., Elbers R.G., Blencowe N.S., Boutron I., Cates C.J., Cheng H.Y., Corbett M.S., Eldridge S.M. (2019). RoB 2: A revised tool for assessing risk of bias in randomised trials. BMJ.

[35] Tufanaru C., Munn Z., Aromataris E., Campbell J., Hopp L. (2020). Chapter 3: Systematic Reviews of Effectiveness. JBI Man. Evid. Synth..

[36] Hoffmann T.C., Glasziou P.P., Boutron I., Milne R., Perera R., Moher D., Altman D.G., Barbour V., Macdonald H., Johnston M. (2014). Better reporting of interventions: Template for intervention description and replication (TIDieR) checklist and guide. BMJ.

[37] Kiechle M., Dukatz R., Yahiaoui-Doktor M., Berling A., Basrai M., Staiger V., Niederberger U., Marter N., Lammert J., Grill S. (2017). Feasibility of structured endurance training and Mediterranean diet in BRCA1 and BRCA2 mutation carriers—An interventional randomized controlled multicenter trial (LIBRE-1). BMC Cancer.

[38] Anderson A.S., Dunlop J., Gallant S., Macleod M., Miedzybrodzka Z., Mutrie N., O’Carroll R.E., Stead M., Steele R.J., Taylor R.S. (2018). Feasibility study to assess the impact of a lifestyle intervention (a € LivingWELL’) in people having an assessment of their family history of colorectal or breast cancer. BMJ Open..

[39] Bruno E., Oliverio A., Paradiso A.V., Daniele A., Tommasi S., Tufaro A., Terribile D.A., Magno S., Filippone A., Venturelli E. (2020). A Mediterranean Dietary Intervention in Female Carriers of BRCA Mutations: Results from an Italian Prospective Randomized Controlled Trial. Cancers.

[40] Vrieling A., Visser A., Hoedjes M., Hurks M., Gómez García E., Hoogerbrugge N., Kampman E. (2018). Increasing awareness and knowledge of lifestyle recommendations for cancer prevention in Lynch syndrome carriers: Randomized controlled trial. Clin. Genet..

[41] Schröder H., Fitó M., Estruch R., Martínez-González M.A., Corella D., Salas-Salvadó J., Lamuela-Raventós R., Ros E., Salaverría I., Fiol M. (2011). A Short Screener Is Valid for Assessing Mediterranean Diet Adherence among Older Spanish Men and Women. J. Nutr..

[42] Fujiyoshi K., Sudo T., Fujita F., Chino A., Akagi K., Takao A., Yamada M., Tanakaya K., Ishida H., Komori K. (2022). Risk of First Onset of Colorectal Cancer Associated with Alcohol Consumption in Lynch Syndrome: A Multicenter Cohort Study. Int. J. Clin. Oncol..

[43] Papadimitriou N., Markozannes G., Kanellopoulou A., Critselis E., Alhardan S., Karafousia V., Kasimis J.C., Katsaraki C., Papadopoulou A., Zografou M. (2021). An umbrella review of the evidence associating diet and cancer risk at 11 anatomical sites. Nat. Commun..

[44] Usher-Smith J.A., Silarova B., Sharp S.J., Mills K., Griffin S.J. (2018). Effect of interventions incorporating personalised cancer risk information on intentions and behaviour: A systematic review and meta-analysis of randomised controlled trials. BMJ Open.

[45] Prochaska J.O., Velicer W.F. (1997). The transtheoretical model of health behavior change. Am. J. Health Promot..

[46] Michie S., van Stralen M.M., West R. (2011). The behaviour change wheel: A new method for characterising and designing behaviour change interventions. Implement. Sci..

[47] Hohberg V., Fuchs R., Gerber M., Künzler D., Paganini S., Faude O. (2022). Blended Care Interventions to Promote Physical Activity: A Systematic Review of Randomized Controlled Trials. Sports Med. Open.

[48] Santarossa S., Kane D., Senn C.Y., Woodruff S.J. (2018). Exploring the Role of In-Person Components for Online Health Behavior Change Interventions: Can a Digital Person-to-Person Component Suffice?. J. Med. Internet Res..

[49] Lavoie A., Dubé V. (2022). Web-Based Interventions to Promote Healthy Lifestyles for Older Adults: Scoping Review. Interact. J. Med. Res..

[50] Long J.C., Winata T., Debono D., Phan-Thien K.C., Zhu C., Taylor N. (2019). Process evaluation of a behaviour change approach to improving clinical practice for detecting hereditary cancer. BMC Health Serv. Res..

[51] Spencer J.C., Wheeler S.B. (2016). A systematic review of Motivational Interviewing interventions in cancer patients and survivors. Patient Educ. Couns..

[52] Teixeira S.M.A., Coelho J.C.F., Sequeira C.A.D.C., Lluch i Canut M.T., Ferre-Grau C. (2019). The effectiveness of positive mental health programs in adults: A systematic review. Health Soc. Care Community.

[53] Chambers S.E., Copson E.R., Dutey-Magni P.F., Priest C., Anderson A.S., Sinclair J.M.A. (2019). Alcohol use and breast cancer risk: A qualitative study of women’s perspectives to inform the development of a preventative intervention in breast clinics. Eur. J. Cancer Care (Engl)..

[54] Anderson A.S., Caswell S., Macleod M., Steele R.J., Berg J., Dunlop J., Stead M., Eadie D., O’carroll R.E. (2017). Health Behaviors and their Relationship with Disease Control in People Attending Genetic Clinics with a Family History of Breast or Colorectal Cancer. J. Genet. Couns..

