# Supplementary material for: Lifestyle Behavior Interventions for Preventing Cancer in Adults with Inherited Cancer Syndromes: Systematic Review
Source: Int J Environ Res Public Health. 2022 Oct 28;19(21):14098. doi: 10.3390/ijerph192114098 (PMC9655661; doi:10.3390/ijerph192114098)
Supplement: Supplementary file 1 [file ijerph-19-14098-s001.zip › ijerph-1936672-supplementary.pdf]

Table S1. PRISMA Checklist of items to include when reporting a systematic review or meta-analysis [29].

Section/topic	Item No	Reported on page No
Title	1	2
Structured summary	2	1
Rationale	3	2-4
Objectives	4	4
Protocol and registration	5	5
Eligibility criteria	6	5-6
Information sources	7	5
Search	8	5 and Table 3
Study selection	9	6
Data collection process	10	6
Data items	11	
Risk of bias in individual studies	12	6
Summary measures	13	6
Synthesis of results	14	6
Risk of bias across studies	15	6
Additional analyses	16	
Study selection	17	7
Study characteristics	18	7
Risk of bias within studies	19	8
Results of individual studies	20	8-9
Synthesis of results	21	8-9
Risk of bias across studies	22	8
Additional analysis	23	9
Summary of evidence	24	9-11
Limitations	25	11
Conclusions	26	12

Table S2. Free-text and MESH terms pertinent to three strings of search. String A was first combined with string B, and subsequently with string C.

A	B	C
Neoplasm (MESH)	"Hereditary cancer"	Risk Reduction Behavio*r
Cancer*	Neoplastic Syndromes, Hereditary (MESH)	Risk Reduction Behavior (MESH)
Neoplas*	Neoplastic Syndromes, Hereditary	Health promotion
Tumo?r*		Health promotion (MESH)
		Healthy Lifestyle
		Healthy Lifestyle (MESH)
		"Lifestyle Intervention"
		"Primary Prevention"
		"Health Behaviour"
		"Cancer Prevention"

Table S3. Example of PubMed search.

(("Neoplasms"[MeSH Terms] OR "cancer*"[All Fields] OR "neoplas*"[All Fields] OR "tumor*"[All Fields] OR "tumour*"[All Fields]) AND ("neoplastic syndromes, hereditary"[MeSH Terms] OR ("neoplastic"[All Fields] AND "syndromes"[All Fields] AND "hereditary"[All Fields]) OR "hereditary neoplastic syndromes"[All Fields] OR ("neoplastic"[All Fields] AND "syndromes"[All Fields] AND "hereditary"[All Fields]) OR "neoplastic syndromes hereditary"[All Fields] OR "neoplastic syndromes, hereditary"[MeSH Terms] OR "hereditary cancer"[All Fields]) AND ("Risk Reduction Behavior"[All Fields] OR "Risk Reduction Behaviour"[All Fields] OR "Healthy Lifestyle"[All Fields] OR "cancer prevention"[All Fields] OR "health behaviour"[All Fields] OR "health behavior"[All Fields] OR "health promotion"[All Fields] OR "primary prevention"[All Fields] OR "healthcare outcomes"[All Fields] OR "lifestyle intervention*"[All Fields] OR "Risk Reduction Behavior"[MeSH Terms] OR "Healthy Lifestyle"[MeSH Terms] OR "health promotion"[MeSH Terms])) AND ((humans[Filter]) AND (english[Filter] OR spanish[Filter]) AND (2010:2023[pdat]))

**Key words**	**PUBMED**
Cancer	S1 Neoplams (Mesh)<br>S2 Cancer*<br>S3 Neoplas*<br>S4 Tumor*<br>S5 Tumour*
Terms above with OR	S1 OR S2 OR S3 OR S4 OR S5: 4477708
Hereditary cancer	S6: “Hereditary cancer”<br>S7 Neoplastic Syndromes, Hereditary (Mesh)<br>S8 Neoplastic Syndromes, Hereditary
Terms above with OR	S6 OR S7 OR S8: 53219
Prevention	S10 Risk Reduction Behavior (Mesh)<br>S11 “Risk Reduction Behavior”<br>S12 “Risk Reduction Behaviour”<br>S13 Healthy Lifestyle (Mesh)<br>S14 “Healthy Lifestyle”<br>S15 “Health Behaviour”<br>S16 “Health Promotion”<br>S17 Health promotion (Mesh)<br>S18 “Healthcare Outcomes”<br>S19 “Lifestyle Intervention”<br>S20 “Primary Prevention”<br>S21 “Cancer Prevention”
Terms above with OR	S10 OR S11 OR S12 OR S13 OR S14 OR S15 OR S16 OR S17 OR S18 OR S19 Or S20 OR S21:238892
Total results with AND	S5 AND S9 AND S20: 791<br>Filters<br>• Language: English Spanish Portuguese<br>• Last 10 years<br>• Humans<br>Total 560
